# Development of on‐shore behavior among polar bears (*Ursus maritimus*) in the southern Beaufort Sea: inherited or learned?

**DOI:** 10.1002/ece3.4233

**Published:** 2018-07-13

**Authors:** Kate M. Lillie, Eric M. Gese, Todd C. Atwood, Sarah A. Sonsthagen

**Affiliations:** ^1^ Department of Wildland Resources Utah State University Logan Utah; ^2^ U.S. Department of Agriculture Wildlife Services National Wildlife Research Center Department of Wildland Resources Utah State University Logan Utah; ^3^ Alaska Science Center U.S. Geological Survey Anchorage Alaska

**Keywords:** climate change, on‐shore behavior, polar bear, social learning, southern Beaufort Sea, *Ursus maritimus*

## Abstract

Polar bears (*Ursus maritimus*) are experiencing rapid and substantial changes to their environment due to global climate change. Polar bears of the southern Beaufort Sea (SB) have historically spent most of the year on the sea ice. However, recent reports from Alaska indicate that the proportion of the SB subpopulation observed on‐shore during late summer and early fall has increased. Our objective was to investigate whether this on‐shore behavior has developed through genetic inheritance, asocial learning, or through social learning. From 2010 to 2013, genetic data were collected from SB polar bears in the fall via hair snags and remote biopsy darting on‐shore and in the spring from captures and remote biopsy darting on the sea ice. Bears were categorized as either on‐shore or off‐shore individuals based on their presence on‐shore during the fall. Levels of genetic relatedness, first‐order relatives, mother–offspring pairs, and father–offspring pairs were determined and compared within and between the two categories: on‐shore versus off‐shore. Results suggested transmission of on‐shore behavior through either genetic inheritance or social learning as there was a higher than expected number of first‐order relatives exhibiting on‐shore behavior. Genetic relatedness and parentage data analyses were in concurrence with this finding, but further revealed mother–offspring social learning as the primary mechanism responsible for the development of on‐shore behavior. Recognizing that on‐shore behavior among polar bears was predominantly transmitted via social learning from mothers to their offspring has implications for future management and conservation as sea ice continues to decline.

## 1 INTRODUCTION

The ability of a species to adapt is fundamental to being resilient to environmental change. A species can biologically respond to change by extinction, shifting its distribution, or adapting to new environmental conditions (Raia, Passaro Fulgione, & Carotenuto, 2012; Teplitsky & Millien, 2014). Alterations in species distribution and abundance that follow shifting of climatic conditions have been documented in several plant and animal species (Parmesan & Yohe, 2003; Root et al., 2003). Similarly, changes in species phenology in response to climate change have been reported (Boutin & Lane, 2014; Charmantier & Gienapp, 2014; Inouye, Barr, Armitage, & Inouye, 2000). Polar bears (*Ursus maritimus*) are experiencing critical and rapid changes to their environment due to climatic warming (Stirling & Derocher, 2012). This ice‐dependent Arctic marine mammal (Amstrup, 2003) was listed as “threatened” under the U.S. Endangered Species Act in 2008 (U.S. Fish and Wildlife Service 2008). The listing was primarily due to the observed and projected loss of sea ice habitat, which puts polar bears at risk of becoming endangered in the foreseeable future (i.e., by mid‐century). During 1979–2014, the spatial extent of Arctic sea ice in September (when sea ice reaches its annual minima) has declined by 13.3% per decade due to warming temperatures (Serreze & Stroeve, 2015). Sea ice extent (and volume) is expected to continue to decline and the southern Beaufort Sea is predicted to become essentially seasonally ice‐free (i.e., <1.0 × 10^6^ km^2^) during the summer before the end of the 21st century (Stroeve et al., 2012). Hence, understanding the ecology and behavior of species dependent on sea ice is necessary for conservation and management actions to ensure their population persistence.

Polar bears depend on sea ice for long‐distance movements, mating, access to their primary prey of ringed seal (*Phoca hispida*) and bearded seal (*Erignathus barbatus*), and some maternal denning (Amstrup, 2003). Numerous studies indicate that survival (Bromaghin et al., 2015; Regehr, Hunter, Caswell, Amstrup, & Stirling, 2010), reproduction, and body condition (Rode, Amstrup, & Regehr, 2010) of the southern Beaufort Sea (SB) subpopulation are negatively affected by changing sea ice conditions. In addition, polar bears have been observed swimming increasingly longer distances as sea ice has, on average, retracted farther from shore during summer (Pilfold, McCall, Derocher, Lunn, & Richardson, 2017), resulting in potentially higher energetic costs (Pagano, Durner, Amstrup, Simac, & York, 2012). Furthermore, the distribution of denning has shifted to include fewer denning sites on the pack ice and more sites on land in correspondence with a reduction in the availability and quality of pack ice serving as denning habitat (Fischbach, Amstrup, & Douglas, 2007).

Polar bears of the SB have historically spent most of the year on the sea ice with the exception of denning (Amstrup, Durner, Stirling, Lunn, & Messier, 2000). However, recent research in Alaska indicates that polar bears of the SB subpopulation are becoming increasingly reliant on land during late summer and fall, when sea ice is no longer present over the biologically productive, shallow water of the continental shelf (Atwood et al., 2016; Gleason & Rode, 2009; Schliebe et al., 2008). The estimated proportion of radio‐collared bears from the SB subpopulation observed on‐shore increased from 5.8% during 1986–1999 to 20.0% during 2000–2014, reaching a peak of 37.0% in 2013 (Atwood et al., 2016).

The number of bears observed on‐shore has been shown to increase when sea ice retracts farther from the shore following the summer melt season (Schliebe et al., 2008). In addition, the spatial distribution of on‐shore bears appears to be linked to the accessibility of ringed seals in off‐shore waters and the availability of subsistence‐harvested bowhead whale (*Balaena mysticetus*) carcasses (Atwood et al., 2016; Schliebe et al., 2008). Coastal Iñupiat communities of Alaska annually hunt bowhead whales and deposit the unused remains at localized “bone piles” on‐shore that consist of trimmed blubber, meat, and bones (Ashjian et al., 2010), thereby attracting polar bears and other wildlife. On‐shore bears could be at a higher risk of human–bear conflicts with local residents, tourists, and industrial activities (Laforge et al., 2017; Wilder et al., 2017), as well as increased exposure to certain pathogens (Atwood et al., 2017) and pollutants (Amstrup, Gardner, Myers, & Oehme, 1989). Despite this marked increase of bears exhibiting on‐shore behavior, there remains a lack of research on how this behavior developed.

Recognizing how animals acquire different behavioral strategies is necessary for both basic and applied scientific disciplines such as wildlife management and conservation biology. Animal behavioral traits can be obtained through genetic inheritance (Arnold, 1981), but frequently the acquisition of a behavior occurs via learning (Heyes, 1994; Heyes & Galef, 1996). Learning incorporates complex ontogenetic processes allowing animals to acquire, store, and use information about the environment (Galef & Laland, 2005). Learning can occur socially or asocially, whereby social learning refers to knowledge acquired from the observation of others, typically a conspecific or the products of their activities, and asocial learning refers to learning where no social interaction is required (Heyes, 1994).

Recent studies have investigated the transmission of foraging behavior from mother to offspring in free‐ranging black bears (*U. americanus*) using observational and genetic techniques (Breck et al., 2008; Hopkins, 2013; Mazur & Seher, 2008). Similarly, studies on grizzly bears (*U. arctos*) examined the transmission of habitat selection and conflict behavior from mother to offspring (Morehouse, Graves, Mikle, & Boyce, 2016; Nielsen, Shafer, Boyce, & Stenhouse, 2013). Bears are good candidates for studying whether particular behaviors are transmitted from mother to offspring because bears are highly intelligent and solitary with the exception of a prolonged mother–offspring association (Gilbert, 1999). Polar bear offspring typically remain with their mother up until 2.3 years of age (Ramsay & Stirling, 1988). Therefore, it is feasible to determine that a bear is learning socially from its mother if bears display the same behavioral patterns as adults.

In light of the pronounced increase in the number of polar bears coming on‐shore and its potential to have both ecological and management implications, our objective was to elucidate how this behavior developed. We collected genetic and behavioral data from bears that come on‐shore (hereafter “on‐shore”) and those that remain on the pack ice (hereafter “off‐shore”) during the fall season. Specifically, we addressed the following question: Was on‐shore behavior for polar bears in the SB subpopulation acquired via asocial learning, social learning, or genetic inheritance?

To answer this question, we tested hypotheses to determine how on‐shore behavior developed via three analyses: (a) genetic relatedness (i.e., quantitative estimate of the proportion of genes shared between the genomes of any two individuals); (b) first‐order relatives (i.e., parent–offspring or sibling pairs); (c) and parentage (i.e., mother–offspring and father–offspring pairs) within and between polar bears categorized as on‐shore and off‐shore bears. We included transmission (i.e., the behavior was transmitted via social learning or genetic inheritance) as an additional hypothesis because not all analyses that we conducted could differentiate between social learning and genetic inheritance. It is important to note that these hypotheses are not mutually exclusive, thus evidence for one hypothesis does not indicate other mechanisms are not occurring but that the most supported hypothesis is more predominant.

### 1.1 Hypothesis 1: On‐shore behavior for polar bears developed via asocial learning

The asocial learning hypothesis from the genetic relatedness analyses predicts that female bears that exhibit on‐shore behavior do not have higher levels of genetic relatedness relative to the entire sampled population. Asocial learning of on‐shore and off‐shore behavior from the parentage analysis would be evident if there was no association between the parent's behavior and the offspring's behavior.

### 1.2 Hypothesis 2: On‐shore behavior for polar bears developed via social learning

The transmission via social learning hypothesis from the genetic relatedness analyses predicts that female bears but not male bears that exhibit on‐shore behavior have higher levels of genetic relatedness relative to the sampled population. Furthermore, an association between the mother's behavior and her offspring's behavior, but no association between the father's behavior and his offspring's behavior (as male bears do not rear cubs), from the parentage analyses would be indicative of social learning for on‐shore and off‐shore behavior.

### 1.3 Hypothesis 3: On‐shore behavior for polar bears developed via genetic inheritance

The transmission via genetic inheritance hypothesis from the genetic relatedness analyses predicts that both female and male bears that display on‐shore behavior have higher levels of genetic relatedness than the sampled population. In addition, a scenario of genetic inheritance of on‐shore and off‐shore behavior from the parentage analyses would be if there was an association between both the mother's behavior and her offspring's behavior and the father's behavior and his offspring's behavior.

### 1.4 Hypothesis 4: On‐shore behavior for polar bears developed via transmission (i.e., social learning or genetic inheritance)

The transmission hypothesis from the genetic relatedness analyses predicts that female bears that exhibit on‐shore behavior have a higher genetic relatedness than the sampled population. Secondly, a higher than expected number of first‐order relatives that display on‐shore behavior would provide evidence of transmission for this behavior.

## 2 MATERIAL AND METHODS

### 2.1 Study area

The SB polar bear subpopulation inhabits a region encompassing areas along the north coast of Alaska and Canada from Icy Cape, USA, (70.3°N, 161.9°W) in the west, to Tuktoyaktuk, Canada (69.4°N, 133.0°W), in the east; following IUCN (Polar Bear Specialist Group; http://pbsg.npolar.no/en/). The southern Beaufort Sea has a narrow continental shelf with a steep shelf‐break that plunges to some of the deepest waters of the Arctic Ocean (Jakobsson et al., 2008). The SB is typically ice covered from October to June, and sea ice retreats to its minimum in the summer and fall seasons from July to September. In recent years there has been a trend in the SB of earlier melt onset, reduced summer sea ice extent, a lengthening of the open‐water season (i.e., sea ice retreats toward the pole during the annual sea ice minimum), and later freeze‐up (Stroeve, Markus, Boisvert, Miller, & Barrett, 2014).

### 2.2 Collection of genetic material

**Figure 1:**
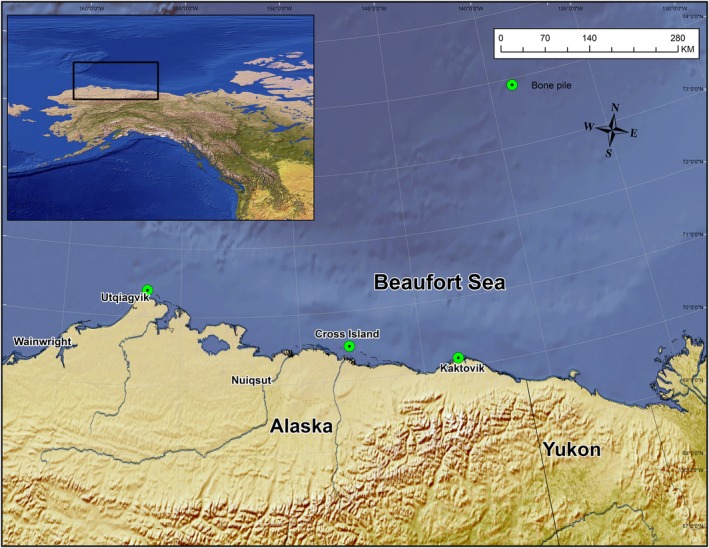
Study area along the Beaufort Sea coast from Utqiagvik, Alaska to the Canadian border

We collected genetic material from SB polar bears from 2010 to 2013 (Figure 1) via direct polar bear captures, remote biopsy darting, and hair snags. We used the contemporary genetic data in conjunction with a long‐term data set of SB polar bears captured nearly every spring since the mid‐1980s. We captured polar bears in coastal areas (e.g., within 150 km of the coast) of the SB from Utqiagvik, Alaska (~157°W) to the U.S.–Canada border (~141°W). We conducted captures over the sea ice during the spring season from March to early May over the study. We encountered adults and subadults opportunistically while flying in a helicopter and immobilized them with tiletamine hydrochloride plus zolazepam hydrochloride (Telazol^®^, Fort Dodge and Warner‐Lambert Co.) using a projectile syringe fired from a dart gun. We collected blood and tissue samples for genetic identification. In addition, we fitted an Argos or global positioning system (GPS) platform transmitter terminal (PTT) satellite radio‐collars to a subset of adult female polar bears to collect movement and spatial data (Durner et al., 2009).

During the spring, we conducted remote biopsy darting from a helicopter to collect tissue samples from adult and subadult bears within approximately 150 km of the coast between Utqiagvik, Alaska, and the U.S.–Canada border. In addition, we conducted remote biopsy darting in the fall along the coastline, barrier islands, and inland areas within approximately 30 km of the coast (Pagano, Peacock, & McKinney, 2014). The remote biopsies collected skin tissue samples for genetic identification. We implemented hair snags in Utqiagvik, Alaska in the fall season of 2011 and Kaktovik, Alaska (~143°W), in the fall seasons of 2012 and 2013 to collect hair samples for genetic identification (see [Herreman & Peacock, 2013] for details).

### 2.3 Genetic identification

We genotyped blood, tissue, and hair samples at 20 microsatellite loci and a ZFX/ZFY sex identification marker by Wildlife Genetics International (Nelson, British Columbia, Canada). The DNA was extracted using a Qiagen DNeasy kit (QIAGEN Inc., Valencia, CA, USA). The DNA was extracted from hair samples using a minimum of 10 guard hair roots, if available, or up to 30 whole underfur hairs if needed to supplement guard hairs. The DNA was extracted from the dry blood and tissue samples using a clipped piece ~3 mm^2^ from the end of a Q‐tip or ear punch. The DNA extracts were initially amplified at 11 hypervariable microsatellite markers to identify individuals: G1A, G10B, G10C, CX110, G1D, G10L, G10M, MU59, G10P (Paetkau & Strobeck, 1994; Proctor, McLellan, & Strobeck, 2002; Taberlet et al., 1997); and G10H and G10J (GenBank accession numbers U22086.1 and U22087.1, respectively). Any DNA extracts that were amplified at <11 loci were considered unsuccessful and excluded from further analyses. After individuals were identified, each individual was amplified at nine additional markers including a sex‐linked locus: MSUT‐2, CPH9, CXX20, MU50, MU51, G10X, CXX173 (Kitahara, Isagi, Ishibashi, & Saitoh, 2000; Molecular Ecology Resources Primer Development Consortium, 2010; Ostrander, Sprague, & Rine, 1993; Paetkau, Calvert, Stirling, & Strobeck, 1995; Proctor et al., 2002; Taberlet et al., 1997); and 14RENP07 and G10U (GenBank accession numbers AJ411284, and U22092.1, respectively).

### 2.4 On‐shore and off‐shore bears

We categorized polar bears based on their behavior as on‐shore or off‐shore individuals both pooled over the duration of the study and on an annual basis. For the pooled data set, we considered bears as on‐shore individuals if they were identified on‐shore during the study. We identified bears on‐shore during the fall season using information from the remote biopsy, hair snag, or GPS locations (see [Atwood et al., 2016] for details). We restricted the fall season to July 1 to October 31 as this was when the sea ice was not contiguous to the coast. We categorized bears as off‐shore individuals if they were identified on the sea ice during the spring remote biopsy or direct capture and were not observed on‐shore at any time during the study. For the annual data set, we considered bears as on‐shore individuals if they were identified on‐shore for a given year from the fall season remote biopsy, hair snag, or GPS locations. We categorized bears as off‐shore individuals if they were not identified on‐shore for that respective year. We conducted the annual categorization because some bears switched behavioral strategies during the 4 years of sampling. We conducted identical analyses on the pooled and the annual data sets to ascertain if comparable results would be obtained.

We estimated the age of individual bears from analysis of cementum annuli (Calvert & Ramsay, 1998), or they were classed as a known age bear if they were originally captured as dependent young with their mother (Ramsay & Stirling, 1988). We conducted three separate analyses to determine how on‐shore behavior was acquired: genetic relatedness, first‐order relatives, and parentage (Breck et al., 2008; Hopkins, 2013). We conducted all statistical tests using α = 0.05 in R (R Core Team 2016). We included an individual bear only once in all analyses after it was categorized as an on‐shore or off‐shore bear. In addition, we included only bears considered independent in all analyses because dependent young had no choice but to remain with their mother. We considered bears as independent if they were ≥2 years old or if they were observed without their mother when captured.

### 2.5 Genetic relatedness

We calculated pairwise relatedness (Queller & Goodnight, 1989) between all possible pairings of individuals using Genalex (Peakall & Smouse, 2006, 2012). Theoretical values of relatedness range from −1 to 1, with negative values indicating the gene frequencies of the two compared individuals differ from the population mean in opposite directions, zero representing random associations between individuals, and increasing values corresponding to increased relatedness. Relatedness values are affected by genetic structure, as these values measure genetic differences in overall allelic frequencies (Queller & Goodnight, 1989). Polar bears are weakly structured throughout their circumpolar distribution (Peacock et al., 2015). No differentiation observed at microsatellite loci among southern Beaufort and adjacent (northern Beaufort and Chukchi Sea) subpopulations was observed; therefore, we conducted analyses among bears across all sampled sites.

We used bootstrap resampling for the genetic relatedness analysis because the relatedness distributions were non‐normal and each behavioral group was a subset of the entire sampled population (Hopkins, 2013). The behavioral groups tested were on‐shore/on‐shore, on‐shore/off‐shore, and off‐shore/off‐shore with mean relatedness determined for the entire sampled population, and females and males, separately. We randomly selected a subset of bears for each behavioral group from the sampled population matrix 10,000 times and calculated relatedness. We then used every relatedness value to generate the bootstrap distribution of the sample mean. We calculated the *p*‐value by the number of times the bootstrap relatedness estimate was greater than or equal to the mean relatedness for the entire sampled population.

### 2.6 Parentage

We identified mother–offspring and father–offspring pairs (Breck et al., 2008) using Cervus 3.0 (Marshall, Slate, Kruuk, & Pemberton, 1998). We considered bears as mothers or fathers if they were estimated to be ≥3 years older than the bear presumed to be the offspring, there were no genotype inconsistencies between parent–offspring pairs, and if parentage assignments were made with ≥80% confidence. We used either a chi‐square goodness‐of‐fit test or a Fisher's exact test (when sample size in at least 1 category was ≤5) to test the null hypothesis that there was no association between the parent's behavior and the offspring's behavior.

### 2.7 First‐order relatives

We used the pairwise relatedness values to identify individual pairs that were first‐order relatives (Breck et al., 2008). Based on relatedness values from known mother–offspring (*n *=* *27) and sibling (*n *=* *6) pairs, we used a value of relatedness ≥0.42 to indicate pairs related at the level of first‐order relatives. We categorized first‐order relatives into the same on‐shore/on‐shore, on‐shore/off‐shore, and off‐shore/off‐shore behavioral groups examined previously. We used either a chi‐square goodness‐of‐fit test or an exact test for multinomial (when sample size in at least 1 category was ≤5) to determine if the number of observed related pairs differed from the number of expected for each behavioral group. We calculated the expected numbers by multiplying the observed number of bears for each behavioral group by the proportion of all possible pairings within a behavioral group.

## 3 RESULTS

A total of 231 independent (i.e., ≥2 years old or if they were observed without their mother when captured) polar bears for the pooled data set were successfully genotyped at a number of loci sufficient to provide individual identity (11) and could be categorized as on‐shore or off‐shore individuals from the behavioral data; of these 123 bears were categorized as off‐shore (59 females and 64 males) and 108 bears were categorized as on‐shore (58 females and 50 males). Over the duration of the study, 12.6% (*n *=* *29/231) of the identified bears switched behaviors among the years. We conducted an annual analysis solely for 2011, because sample size for independent bears was the highest (2010: *n *=* *81, 2011: *n *=* *103, 2012: *n *=* *97, 2013: *n *=* *57) and we had sufficient data for mother–offspring and father–offspring pairs to conduct the Fisher's exact test. In 2011, there were 103 identified independent bears with behavioral data; we categorized 47 bears as off‐shore (24 females and 23 males) and 56 bears as on‐shore (28 females and 28 males).

Female on‐shore/on‐shore pairs had the highest mean relatedness of all behavioral groups (Table 1), which was significantly higher than the mean relatedness of the entire sampled population. Male on‐shore/on‐shore pairs did not have a significantly higher mean relatedness than the mean relatedness of the entire sampled population, which provided evidence of social learning of on‐shore behavior given that the female on‐shore/on‐shore pairs had significantly higher relatedness than the sampled population. A similar pattern was observed for the 2011 annual analysis. Among the 2011 analyses, only female on‐shore/on‐shore pairs had significantly higher mean relatedness than the mean relatedness for the entire sampled population (and the highest mean relatedness of all behavioral groups). In contrast, male on‐shore/on‐shore pairs did not have a significantly higher mean relatedness than the mean relatedness of the entire sampled population.

**Table 1:** Mean relatedness and corresponding *p*‐values of behavioral groups by category for polar bears of the southern Beaufort Sea, pooled for 2010–2013 and annually for 2011. The *p*‐value was calculated by the number of times the bootstrap relatedness estimate for each behavioral category was greater than or equal to the mean relatedness for the entire sampled population

Behavioral groups	*n*	Mean relatedness	*p*‐value
Pooled			
Sampled population	231	−0.0043	
On‐shore/on‐shore		0.0066	0.082
On‐shore/off‐shore		−0.0075	0.726
Off‐shore/off‐shore		−0.0072	0.648
Female bears	117		
On‐shore/on‐shore		0.0151	0.039
On‐shore/off‐shore		−0.0005	0.298
Off‐shore/off‐shore		−0.0020	0.406
Male bears	114		
On‐shore/on‐shore		−0.0018	0.406
On‐shore/off‐shore		−0.0141	0.904
Off‐shore/off‐shore		−0.0147	0.849
2011			
Sampled population	103	−0.0098	
On‐shore/on‐shore		−0.0007	0.192
On‐shore/off‐shore		−0.0129	0.657
Off‐shore/off‐shore		−0.0151	0.678
Female bears	52		
On‐shore/on‐shore		0.0110	0.089
On‐shore/off‐shore		−0.0196	0.810
Off‐shore/off‐shore		−0.0109	0.524
Male bears	51		
On‐shore/on‐shore		−0.0083	0.459
On‐shore/off‐shore		−0.0241	0.894
Off‐shore/off‐shore		−0.0079	0.458

There was evidence of an association between a mother's behavior and her offspring's behavior (Table 2). The numbers of on‐shore/on‐shore and off‐shore/off‐shore mother–offspring pairs were higher than expected. The number of on‐shore/off‐shore mother–offspring pairs was lower than expected consistent with the pattern of offspring retaining the behavioral strategy of their mother. The same pattern was observed for the 2011 data set, though the signal was not as strong. The number of on‐shore/on‐shore and off‐shore/off‐shore mother–offspring pairs was higher than expected, while the number of on‐shore/off‐shore mother–offspring pairs was lower than expected. There was no significant association between a father's behavior and his offspring's behavior (Table 3) for the pooled data set or for the 2011 data set; though the sample size was low for 2011 and may limit the power of the test. Collectively, the parentage findings provide evidence for mother–offspring social learning of on‐shore behavior.

**Table 2:** Observed and expected mother–offspring pairs by behavioral group for polar bears of the southern Beaufort Sea, pooled for 2010–2013 and annually for 2011. A chi‐square goodness‐of‐fit test or a Fisher's exact test (when sample size in at least 1 category was ≤5) was used to test the null hypothesis that there is no association between the parent's behavior and the offspring's behavior

Relationship	Observed	Expected	*p*‐value
Pooled			
Mother–offspring			
On‐shore/on‐shore	32	28	0.004
Off‐shore/on‐shore	4	8	
On‐shore/off‐shore	6	10	
Off‐shore/off‐shore	7	3	
2011			
Mother–offspring			
On‐shore/on‐shore	14	13	0.056
Off‐shore/on‐shore	1	3	
On‐shore/off‐shore	1	3	
Off‐shore/off‐shore	2	1	

**Table 3:** Observed and expected father–offspring pairs by behavioral group for polar bears of the southern Beaufort Sea, pooled for 2010–2013 and annually for 2011. A chi‐square goodness‐of‐fit test or a Fisher's exact test (when sample size in at least 1 category was ≤5) was used to test the null hypothesis that there is no association between the parent's behavior and the offspring's behavior

Relationship	Observed	Expected	χ^2^	*p*‐value
Pooled				
Father–offspring				
On‐shore/on‐shore	17	15	0.8755	0.349
Off‐shore/on‐shore	7	9		
On‐shore/off‐shore	7	9		
Off‐shore/off‐shore	7	5		
2011				
Father–offspring				
On‐shore/on‐shore	3	2	0.0521	0.400
Off‐shore/on‐shore	0	1		
On‐shore/off‐shore	1	2		
Off‐shore/off‐shore	1	0		

The observed number of first‐order relatives deviated from the expectation for both the pooled and 2011 data sets (Table 4). The number of on‐shore/on‐shore first‐order relatives was higher than expected, which provided evidence for transmission via genetic inheritance or social learning of on‐shore behavior. Conversely, the number of on‐shore/off‐shore and off‐shore/off‐shore first‐order relatives was lower than expected.

**Table 4:** Observed and expected first‐order relatives by behavioral group for polar bears of the southern Beaufort Sea, pooled for 2010–2013 and annually for 2011. A chi‐square goodness‐of‐fit test or an exact test for multinomial (when sample size in at least 1 category was ≤5) was used to determine whether the number of observed related pairs differed from the number of expected for each behavioral group

Behavioral groups	Observed	Expected	χ^2^	*p*‐value
Pooled				
On‐shore/on‐shore	64	25	80.8917	<0.001
On‐shore/off‐shore	30	57		
Off‐shore/off‐shore	19	32		
2011				
On‐shore/on‐shore	21	8	33.2949	<0.001
On‐shore/off‐shore	3	13		
Off‐shore/off‐shore	2	5		

## 4 DISCUSSION

Analyses testing relationships based on genetic relatedness and parentage estimates revealed that social learning was the primary mechanism responsible for on‐shore behavior. This was revealed by the finding that the female on‐shore/on‐shore behavioral category had a significantly higher mean relatedness than the entire sampled population, while the male on‐shore/on‐shore behavioral category did not (Table 1). Thus, female polar bears exhibiting on‐shore behavior had higher relatedness; while on‐shore males were not more related than the general population. Furthermore, a significant association between a mother's behavior and her offspring's behavior was observed (Table 2), while no association between a father's behavior and his offspring's behavior was found (Table 3). In combination, the parentage results indicated that the transmission of on‐shore and off‐shore behavior was through mother–offspring social learning because independent offspring generally continued to follow the same behavioral strategy of their mother.

All three analyses from both the pooled and annual data sets suggested transmission, via social learning or genetic inheritance, of on‐shore behavior for the SB polar bear subpopulation. The pooled and annual data sets had concordant results indicating that bears switching behavior among the years did not alter the overall conclusions. Analysis based on first‐order relatives revealed higher than expected on‐shore/on‐shore first‐order relatives and lower than expected on‐shore/off‐shore and off‐shore/off‐shore first‐order relatives (Table 4). Close relatives exhibiting the same behavior indicated transmission of on‐shore behavior because closely related individuals were likely socially learning from each other or there was a genetic basis for on‐shore behavior.

A high proportion of male polar bears leaving the study area could have resulted in similar patterns in our genetic relatedness analysis; thereby erroneously producing a signature of social learning. For example, male grizzly bears travel widely during breeding season (Ciarniello, Boyce, Seip, & Heard, 2007) and generally have longer natal dispersal distances than females (McLellan & Hovey, 2001; Proctor, McLellan, Strobeck, & Barclay, 2004), which would likely result in a higher level of genetic relatedness among female bears in a region. Generally, movements of male and female polar bears do not differ greatly (Amstrup, Durner, McDonald, Mulcahy, & Garner, 2001) but female polar bears can have larger breeding range sizes than males (Laidre et al., 2013); whereas Zeyl, Aars, Ehrich, and Wiig (2009) found that polar bears of the Barents Sea exhibit male‐biased natal dispersal. Thus, because dispersal distance is sex‐biased in polar bears, the scenario of higher genetic relatedness among female bears exhibiting on‐shore behavior could be a result of greater male dispersal. Nonetheless, the mother–offspring findings provided evidence of social learning despite the uncertainty regarding the genetic relatedness results because offspring generally followed the same behavioral strategy as their mother.

Lower survival of off‐shore polar bears could also generate equivalent results. That is, if on‐shore bears have higher survival, and therefore on‐shore females have a higher recruitment rate of cubs than off‐shore bears, then higher genetic relatedness among on‐shore bears, a higher proportion of on‐shore/on‐shore first‐order relatives, and more on‐shore/on‐shore mother–offspring pairs would be observed. Thus far, no studies have been conducted on survival and recruitment comparing on‐shore and off‐shore polar bear subpopulations. However, research on SB polar bears found similar activity patterns and physiological condition for on‐shore and off‐shore bears, which suggests that neither the on‐shore or off‐shore group realizes a greater benefit than the other (Whiteman et al., 2015). While the mother–offspring data suggest on‐shore behavior was acquired through social learning, we cannot rule out the possibility that off‐shore mothers experienced a high incidence of reproductive failure, which then contributed to the clustering of relatives on‐shore.

Behavioral or physiological modifications in response to climate‐driven changes in their environment have been observed in other species (Bradshaw & Holzapfel, 2006) with both positive and negative fitness consequences (Both, Bouwhuis, Lessells, & Visser, 2006; Halupka, Dyrcz, & Borowiec, 2008; Réale, McAdam, Boutin, & Berteaux, 2003). The increase in SB polar bears coming on‐shore (Atwood et al., 2016) and the transmission of this behavior via mother–offspring social learning may be a behavioral modification in response to climate change and suggests that some SB polar bears are altering their behavior in response to a changing climate. Furthermore, some bears were observed switching behaviors over the duration of the study revealing that these behaviors are dynamic. Bears may alter their behavior for a multitude of reasons, such as annual sea ice conditions, food availability, and reproductive status. Plasticity in on‐shore/off‐shore behavior may provide an avenue for polar bears to respond to changing sea ice conditions on an annual basis.

On‐shore bears may be exposed to additional risks, including a greater potential for human–bear conflicts and increased exposure to contaminants and diseases (Stirling & Derocher, 2012). There are several villages along the north coast of Alaska and an industrial footprint associated with oil exploration and extraction, all of which can occur in relatively close proximity to on‐shore bears. Also in close proximity to human settlements are the remains of subsistence‐harvested bowhead whale carcasses, which are deposited on land and attract large aggregations of bears (Herreman & Peacock, 2013). Therefore, human–bear conflicts will likely increase as the sea ice continues to decline and more bears come ashore. Human–wildlife conflicts can have broad effects: negatively impacting wildlife populations, changing the structure of ecosystems (Woodroffe, Thirgood, & Rabinowitz, 2005), and endangering public safety (Thirgood, Woodroffe, & Rabinowitz, 2005). Other polar bear populations, such as the Western Hudson Bay population, have experienced increases in the number of problem bears correlated with delayed sea ice formation and changes in polar bear distribution and declining body condition. In addition, polar bears that were highly motivated to obtain food appeared more willing to risk interacting with humans (Towns, Derocher, Stirling, Lunn, & Hedman, 2009).

The proportion of SB polar bears exhibiting on‐shore behavior during the fall season has increased over time (Atwood et al., 2016; Pongracz & Derocher, 2016). Furthermore, trends of earlier arrival on‐shore, increased length of stay, and later departure back to the sea ice have been detected, which are all related to declines in the availability of sea ice habitat over the continental shelf and changes to sea ice phenology. The Arctic is expected to continue to warm given the current trends in global greenhouse emissions (Larsen et al., 2014). Thus, SB polar bears will likely continue to experience changes to their environment resulting in more bears coming on‐shore. Therefore, it will be important to monitor the population‐level consequences of extended land use. Properly managing polar bear mother–offspring pairs, when feasible, will be important to ensure their continued persistence in a rapidly changing environment and mitigate human–bear conflicts for this apex predator in the changing Arctic.

## CONFLICT OF INTEREST

None declared.

## AUTHORS’ CONTRIBUTIONS

All authors conceived and designed the study. T.A. carried out field studies. K.L. then analyzed the data and drafted the manuscript, with all authors contributing to revisions.
